# Large-scale fabrication of ordered arrays of microcontainers and the restraint effect on growth of CuO nanowires

**DOI:** 10.1186/1556-276X-6-86

**Published:** 2011-01-17

**Authors:** Pengrui Shao, Shaozhi Deng, Jun Chen, Ningsheng Xu

**Affiliations:** 1 State Key Laboratory of Optoelectronic Materials and Technologies, Guangdong Province Key Laboratory of Display Material and Technology, School of Physics and Engineering, Sun Yat-sen University, Guangzhou 510275, People's Republic of China

## Abstract

Technique has been developed to fabricate ordered arrays of microcontainers. We report that ordered microcontainer arrays of Cu can be fabricated on glass substrate by thin film deposition and self-assembly technology. In addition, CuO nanowires are found to grow only in the inner sides of microcontainers, which verifies the stress growth mechanism of CuO nanowires. High-resolution transmission electron microscopy study reveals that CuO nanowires grow along the [110] direction. Such structure may have potential application in micro-electron sources, which have the self-focused function.

## Introduction

Fabrication of arrays of three-dimensional (3D) micro- or nanostructures is one of the challenging tasks [1,2]. Much effort has been made to study their fabrication and potential applications such as in biosensor [3], lithium secondary batteries [4], and micro- or nanocontainers for reaction. Wang et al. [5] fabricated large-scale ordered arrays of TiO_2 _nanobowl by utilizing monolayer self-assembly and atomic layer deposition. Zhang et al. [6] used colloidal crystals template to fabricate 3D ordered macroporous rare-earth oxides and Li et al. [7] reviewed similar ways for preparation of various ordered micro- or nanostructured arrays. Srivastava et al. [8] developed a modified infiltration approach for the fabrication of arrays of cobalt nanobowl. Wang et al. [9] made free-standing ZnO nanobowls. Kim et al. [10] investigated formation process of the polypyrrole microcontainers. Zhan et al. [11] investigated the anomalous infrared transmission of gold films on 2D colloidal crystals. Ye et al. [12] carried out fabrication, characterization, and optical property study of gold nanobowls. However, most of the above micro- or nanostructures have been achieved by the top-down method.

Here, technique based on self-assembly has been developed. Ordered arrays of microcontainers of copper oxide have been fabricated in large-scale and CuO nanowires have been found to grow only in the inner sides of the microcontainers without use of any catalysts. Moreover, this general and facile method can be applied to fabricate the similar 3D structures using other metals (such as Zn, Cr, Fe, etc.) and/or their oxides microcontainers.

### Experimental section

**Figure 1:**
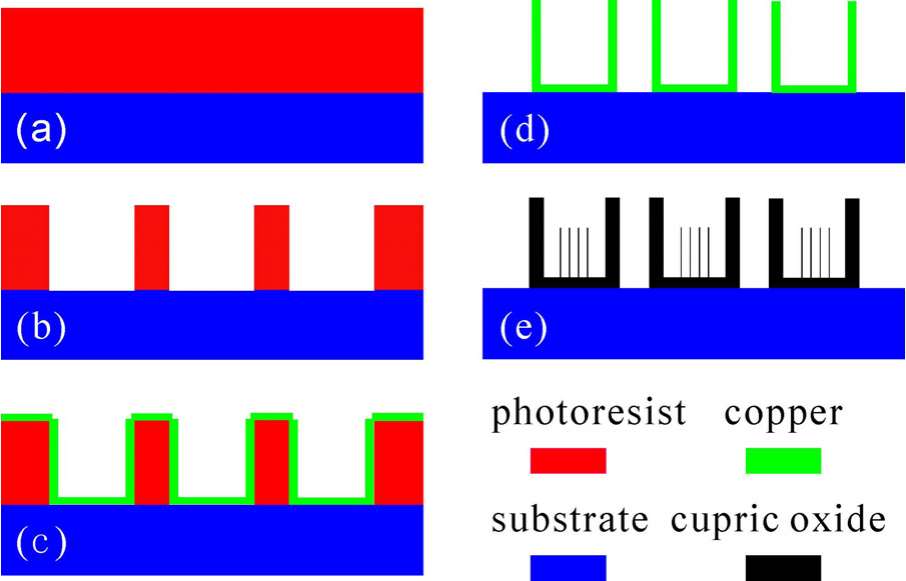
**Schematic description of the fabrication processes**. **(a) **Photoresist layer spin-coated on substrate, **(b) **UV exposure and development of the photoresist, **(c) **deposition of copper layer by DC sputtering, **(d) **removing of photoresist, and **(e) **growth of CuO nanowires by thermal oxidation.

The fabrication process of the microcontainers is illustrated in Figure 1. The glass substrate of 1.1 mm in thickness is first washed by using liquid soap solution and sequentially cleaned for 10 min in an ultrasonic bath of acetone, ethanol, and deionized water, respectively. Finally, it is dried by nitrogen flow. Then, a layer of positive photoresist (RZJ-390) of 2.5 μm in thickness is spined on glass substrate (Figure 1a) and subsequently exposed to UV light through a mask (Figure 1b). Cu thin film of 400 nm in thickness is then deposited by DC sputtering (Figure 1c). Cu thin film and photoresist are peeled off using acetone, shown in Figure 1d. Finally, CuO nanowires grow in a self-assembly process by thermal oxidation of ordered arrays of the microcontainers of Cu at 400°C for 3 h in air. The morphology and structure of the as-prepared samples are investigated by field emission scanning electron microscope (FE-SEM, Quanta 400F) and high-resolution transmission electron microscopy (JEM-2010HR).

## Results and discussion

**Figure 2:**
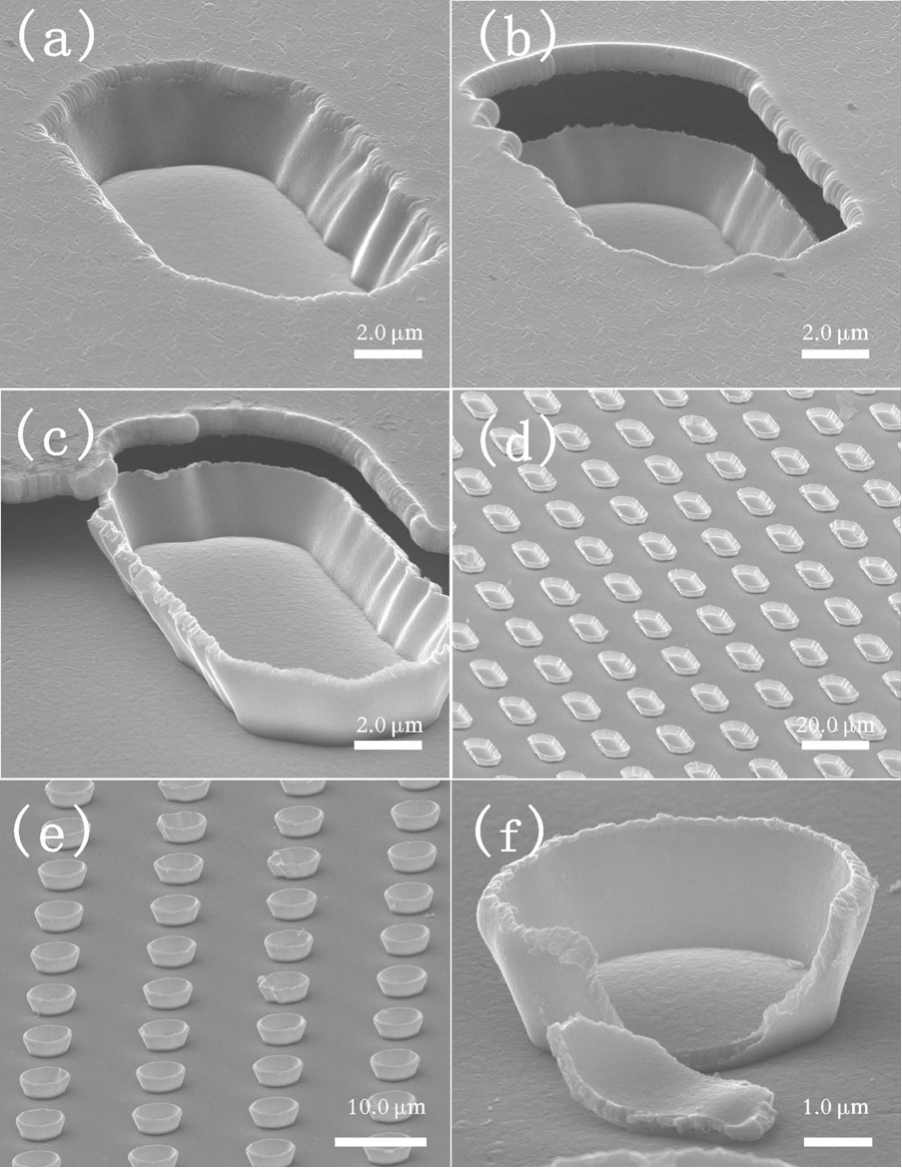
**SEM images (70° oblique view) showing the formation process of Cu microcontainer arrays**; (**a**) deposition of Cu film, (**b**) and (**c**) photoresist dissolved by acetone during the peeling off, (**d**) microboat array after completely removing of photoresist,(**e**) microbowl arrays, (**f**) single high magnification microboat.

Figure 2a,b,c clearly show the formation process of array of microboats of Cu on glass substrate. The wall thickness of microboats is dependent on the thickness of the deposited film, while the height is dependent on the thickness (*l*_1_) of the coated photoresist and the thickness (*l*_2_) of the deposited Cu film: *h *= *l*_1_-*l*_2_. Figure 2d shows an array of Cu microboats. Figure 2e shows an array of Cu microbowls with a high magnification SEM image of one of the microbowls being shown in Figure 2f. From Figure 2f, we can see the wall thickness of microbasins is 400 nm.

**Figure 3:**
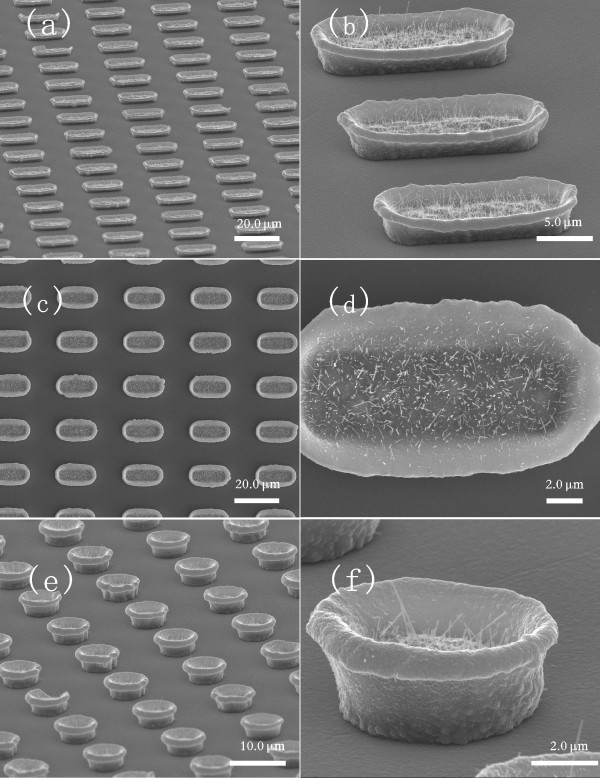
**CuO nanowires grown in microboats and microbowls by thermal oxidation in air**; **(a, b) **70° oblique views, **(c, d) **top views for microboats, and **(e, f) **70° oblique views for microbowls.

Figure 3 shows arrays of CuO microboats and microbowls containing CuO nanowires, which grew in a self-assembly process by thermal oxidation of Cu microboats and microbowls. Comparing with those shown in Figure 2, edges of microboats and microbowls have become thicker after the thermal oxidation process. It is noticeable that CuO nanowires grew only in the inner surface of the microboats and microbowls. Their diameters are 30-80 nm and length 0.5-4 μm.

**Figure 4:**
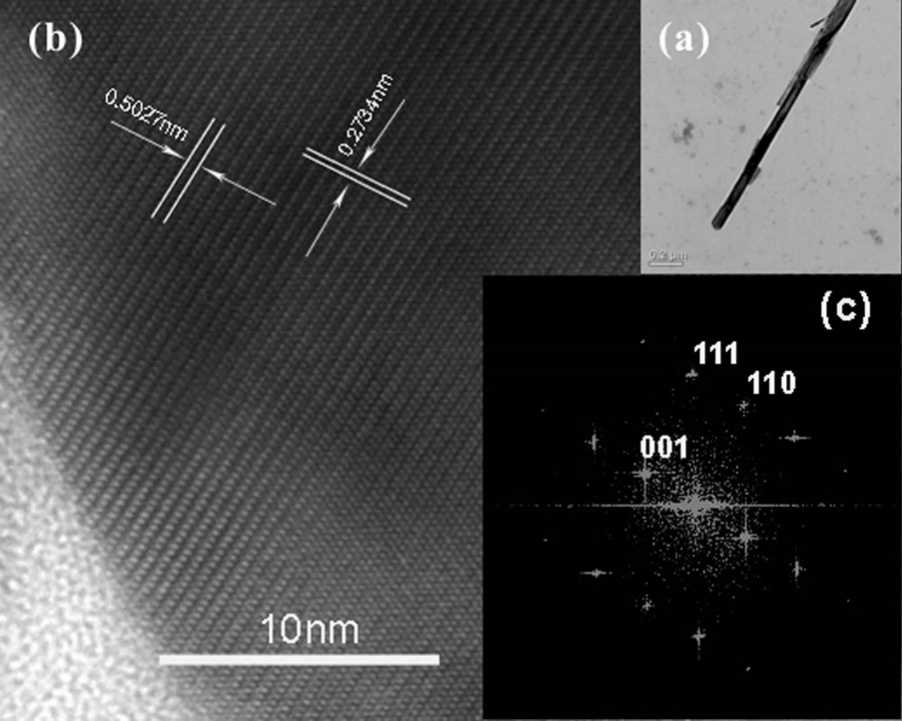
**TEM images of one single CuO naonwire**; (**a**) TEM image, (**b**) corresponding HRTEM image and (**c**) a power spectrum made by Fourier transforming the HRTEM image.

The microstructure of the individual CuO nanowires was further examined using TEM. Figure 4a shows a typical TEM image of a CuO nanowire. A typical HRTEM image of a single nanowire is given in Figure 4b, and the clearly visible fringes reveal that the nanowire is crystalline. The distance between the crystal face is about 0.2734 nm, which corresponds to the {110} plane. A power spectrum made by Fourier transforming the HRTEM image in Figure 4c indicates that the CuO nanowire is monoclinic type. This also proves that the growth direction of CuO nanowires is along the [110] direction.

Different growth mechanism of CuO nanowires has been proposed by different research groups. Jiang et al. [13] believe that the formation of CuO nanowires by thermal oxidation obeys vapor-solid model (VS), where the growth of CuO nanowires depends on different vapor pressure of CuO. Liu et al. [14] have proposed a base-up self-diffusion model; namely, the growing process of CuO nanoneedles is controlled by the diffusion of the copper ions from the substrate, which is caused by the local electrical field set up by the oxygen ions at the solid/gas interface. Kaur et al. [15] and Kummar et al. [16] have attributed the formation of CuO nanowires to relaxation of accumulating stress. According to the VS mechanism, there exist CuO nanowires on the outside surface of microcontainers in our case. However, we do not observe any CuO nanowires on the outer surface of microcontainers. We believe that the growth of CuO nanowires is due to compressive stress. During the oxidation of Cu microcontainer, oxygen ions will diffuse inside the Cu film. Then a layer of CuO will form on both outer and inner surface of Cu microcontainer, which leads to volume expansion of microcontainer. But the CuO film cannot expand along the surface, because the film is relatively compact. The CuO film can only expand along normal direction of the surface. Due to space limit, CuO film on the inner surface of microcontainer will become concentrated as expansion, while the film on the outer of microcontainer become scattered. Therefore, compressive stress at the inner surface will become greater and greater during oxidation, and finally lead to growth of nanowires. While there is tensile stress at the outer surface, no nanowires can be grown.

**Figure 5:**
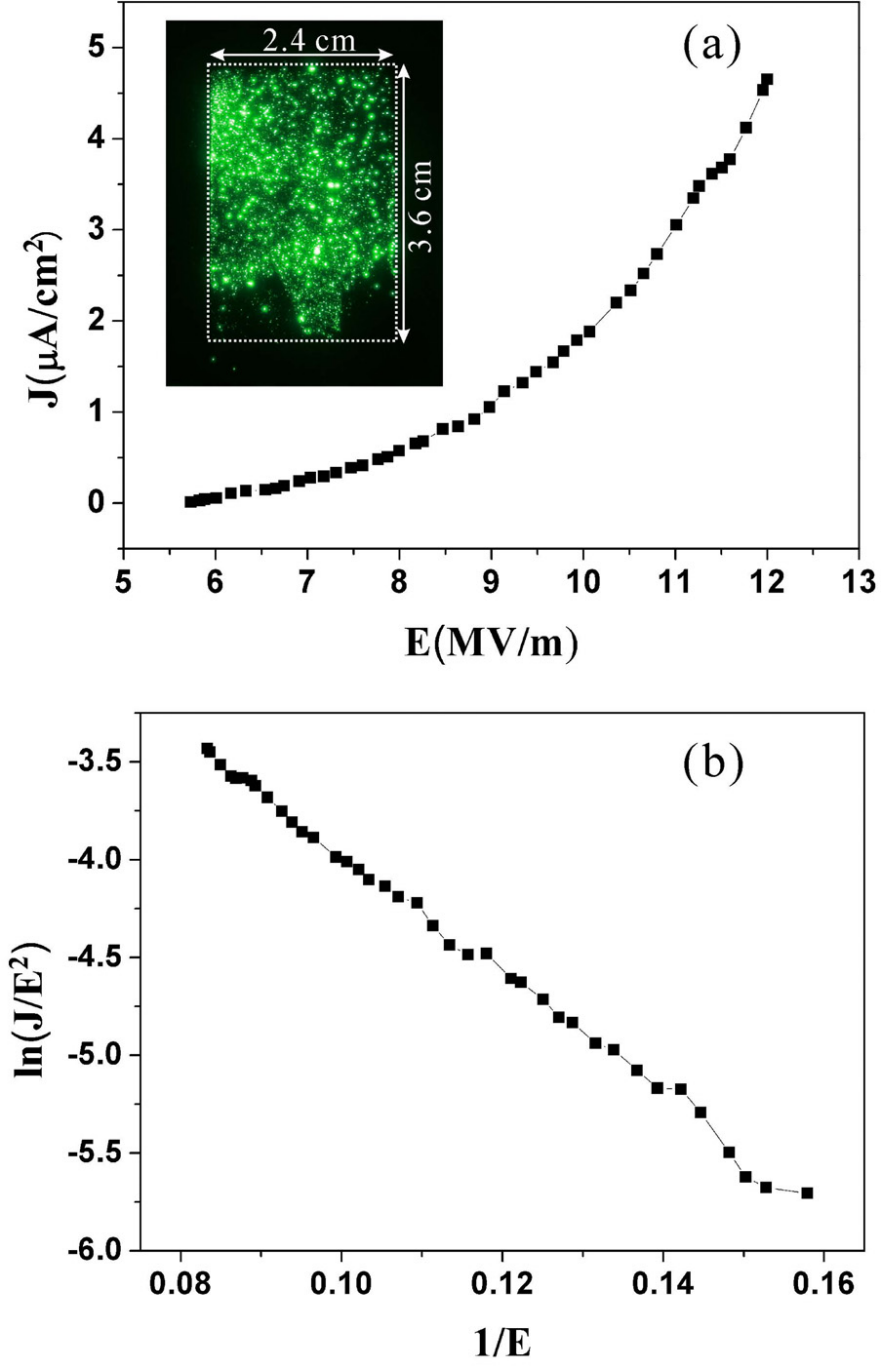
**Field electron emission characteristics of the CuO nanowires grown in microboats**. **(a) ***J*-*E *plots and emission images (inset) and **(b) **the corresponding F-N plots.

**Figure 6:**
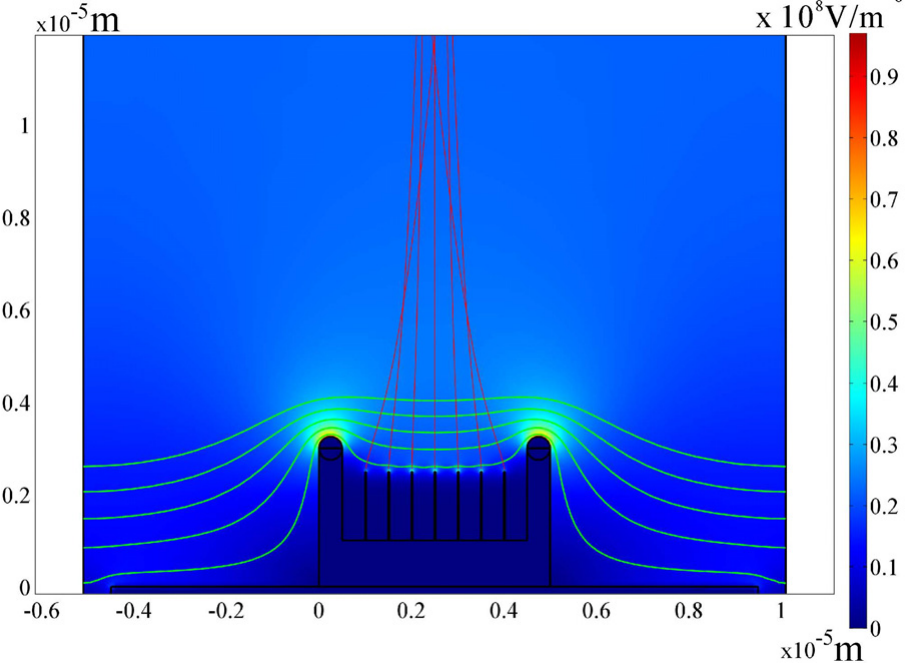
**Simulation of traces of electron emitted from nanowires, showing effect of self-focused by the microcontainer**. The red lines are the traces of electron.

To investigate the field emission characteristics of CuO nanowires grown in arrays of microboats, green phosphor (ZnS)-coated indium tin oxide glass, kept at a distance of 250 μm from the sample surface, was used as an anode in a diode-type configuration. Figure 5 shows the typical field emission characteristics measured under a base vacuum of 2.4 × 10^-5 ^Pa. The current density (*J*) increases the applied electric field (*E*). As shown in the emission image of inset of Figure 5a, it is obviously seen that anode voltage can effectively induce electron emission from CuO nanowire grown in microboats. The corresponding FN plots exhibit linearity shown in Figure 5b. The possible application of CuO nanowires grown in microcontainers includes self-focused electron sources. In field emission display (FED), especially micro-display, for example, gated structure's FED, trajectories of emitted electrons are often divergent because of nonuniform electric field formed by gate voltage. This effect reduces display resolution especially in microdisplay device. In our microcontainers, electrons can be focused, which will improve display resolution as shown in Figure 6. This effect needs further dedicated experimental study.

## Conclusion

In conclusion, we have demonstrated a versatile method to fabricate ordered arrays of metallic or its oxide microcontainers. Growth of CuO nanowire is observed to be retrained by the Cu microcontainers because of compressive stress accumulation. The HRTEM study reveals that CuO nanowires grow along the [110] direction. A potential application of the microcontainers in practical devices is also simulated. Related experiments for application of 3D metallic/oxide microcontainers, such as using vacuum electron sources, batteries, etc., need to be investigated in future.

## Competing interests

The authors declare that they have no competing interests.

## Authors' contributions

PS carried out the fabrication of microcontainers, and drafted the manuscript. JC carried out the field emission test. SD participated in the design of the study and discussion of growth mechanism of CuO nanowires. NX participated in the design of the study, and critically revised the manuscript for important intellectual content, and has given final approval of the version to be published. All authors read and approved the final manuscript.
